# Health related quality of life during dialysis modality transitions: a qualitative study

**DOI:** 10.1186/s12882-023-03330-y

**Published:** 2023-09-22

**Authors:** Chance S. Dumaine, Danielle E. Fox, Pietro Ravani, Maria J. Santana, Jennifer M. MacRae

**Affiliations:** 1 Division of Nephrology, University of Saskatchewan, Saskatoon, SK Canada; 2 Department of Community Health Sciences, University of Calgary, Calgary, Canada; 3 Cumming School of Medicine, University of Calgary, Calgary, Canada; 4 Division of Nephrology, Cumming School of Medicine, University of Calgary, Calgary, Canada; 5 Department of Cardiac Sciences, University of Calgary, Calgary, Canada; 6 Department of Pediatrics, University of Calgary, Calgary, Canada

**Keywords:** Transitions, Health related quality of life, Kidney failure, Dialysis

## Abstract

**Background:**

Modality transitions represent a period of significant change that can impact health related quality of life (HRQoL). We explored the HRQoL of adults transitioning to new or different dialysis modalities.

**Methods:**

We recruited eligible adults (≥ 18) transitioning to dialysis from pre-dialysis or undertaking a dialysis modality change between July and September 2017. Nineteen participants (9 incident and 10 prevalent dialysis patients) completed the KDQOL-36 survey at time of transition and three months later. Fifteen participants undertook a semi-structured interview at three months. Qualitative data were thematically analyzed.

**Results:**

Four themes and five sub-themes were identified: adapting to new circumstances (tackling change, accepting change), adjusting together, trading off, and challenges of chronicity (the impact of dialysis, living with a complex disease, planning with uncertainty). From the first day of dialysis treatment to the third month on a new dialysis therapy, all five HRQoL domains from the KDQOL-36 (symptoms, effects, burden, overall PCS, and overall MCS) improved in our sample (i.e., those who remained on the modality).

**Conclusions:**

Dialysis transitions negatively impact the HRQoL of people with kidney disease in various ways. Future work should focus on how to best support people during this time.

**Supplementary Information:**

The online version contains supplementary material available at 10.1186/s12882-023-03330-y.

## Background

Globally, the number of people on dialysis is increasing, with North America having one of the highest rates [1]. Dialysis utilization not only uses a disproportionate amount of healthcare resources [2] but can be burdensome to people on dialysis. Despite people on dialysis reporting a lower health related quality of life (HRQoL) compared to the general population [3, 4], studies that have been conducted to explore HRQoL across dialysis modalities have not consistently shown a difference between modality types (e.g., hemodialysis, peritoneal dialysis, home hemodialysis) [5]. This may partially be explained by the dynamic changes to HRQoL throughout dialysis care trajectories [6], making it important to target times in care trajectories where HRQoL needs are high regardless of dialysis modality.

Dialysis transitions and changes in modality have been highlighted as a time where attention to the potential changes in HRQoL should be considered [7–9]. Transition periods in dialysis care pathways can be especially tumultuous [10]. People embark on a new way of life, challenging their coping mechanisms and making them re-evaluate their identities, their relationships with themselves, others, and with their disease and treatment [10]. Kidney failure transition periods are often triggered by declining health and medical necessity, and have been described as emotionally turbulent, “marked by periods of emotional upheaval and doubts about the future” [10, 11]. In addition to being times of mental and emotional stress, transition periods are also often periods of physical stress as people may be experiencing several symptoms related to uremia or inadequate dialysis. Morbidity and mortality have also been shown to increase during dialysis transitions, [12, 13] which may further impact HRQoL. Very few studies examine the changes in HRQoL in the transition period [3, 14, 15] and of these most compare the prospective changes in HRQoL from prior to dialysis initiation to 6 or 12 months later [3, 15].Unfortunately there is a paucity of literature on how HRQoL is impacted during the early dialysis transition, which is needed to better understand how kidney programs can support people during this time. We thus aimed to explore the HRQoL experiences of adults who were transitioning to a new dialysis modality.

## Methods

### Setting

This study was conducted in a large urban city in Western Canada that has seven adult dialysis sites that collectively serve approximately 680 patients on dialysis, with approximately 25 patients transitioning to dialysis every month. The three sites where patients primarily transition to dialysis were selected for recruitment and include an in-center hemodialysis (IHD) center, a home hemodialysis (HHD) clinic, and a peritoneal dialysis (PD) clinic.

### Participants

Eligible adults attending an in-person appointment at the dialysis clinic during our study period (July 2017 to September 2017) were informed about the study by an intermediary. Interested individuals were approached by a member of the research team. Those who agreed to participate provided written informed consent and completed a baseline survey and demographic questionnaire. Participants were then contacted by telephone for later data collection phases. We included adults (≥ 18) who were undergoing a planned transition to dialysis or undertaking a modality change. As people undergoing temporary transitions may have different experiences, we excluded those who had an acute kidney injury and/or were expected to recover kidney function, and those who were undergoing a temporary modality change anticipated to last less than three months. We further excluded people who were not English speaking due to the lack of resources required to hire an interpreter and translate the study data.

### Data collection

We leveraged the Kidney Disease Quality of Life-36 (KDQOL-36) to guide data collection. The KDQOL-36 is a validated tool for both pre-dialysis and dialysis patients that assess HRQoL in two generic domains [physical component summary score (PCS) and mental component summary score (MCS)] and three kidney disease-specific domains (“Symptoms/Problems”, “Burden of Kidney Disease”, and “Effects of Kidney Disease on Daily Life”) [16–18].

A male graduate student (CD), with one year of experience and training in qualitative research and no prior relationship with the participants, conducted semi-structured telephone interviews approximately three months after starting on their new modality. The interview guide was developed to collect qualitative data to complement the five domains of the KDQOL-36. The open-ended questions allowed patients to elaborate and provide context for how their modality transition impacted different aspects of their quality of life. (Supplementary File 1). Telephone based interviews were used as they are an effective way to increase participant comfort and allow participants to be more forthcoming with responses [19–21]. Participants who could not be contacted by telephone were approached at their next clinic visit. All participants were asked the same guiding questions, however, probing questions changed based on participant responses and queries derived from past interviews. All interviews were audio recorded, transcribed verbatim, and fieldnotes were taken. The transcripts were not returned to participants for approval.

To further explore HRQoL, participants completed an initial paper survey comprised of the KDQOL-36 survey domains within the first week of their modality transition and typically on their first day of IHD or their first day of home dialysis training (PD and HHD) and again three months later. The survey was most often filled out in private but if the patient requested it, a family member was present. Patients who were not coming to the clinic were mailed the surveys and asked to return them in a sealed envelope at their next clinic appointment.

### Data analysis

We conducted an inductive thematic analysis to analyze the interview data [22, 23]. Interviews were transcribed verbatim. CD, DF, and JM then read the transcripts through several times to immerse in the data. An initial coding framework was generated through discussion and open coding of the dataset. Codes were refined, named, and defined through iterative rounds of discussion and further coding until distinct overarching themes were formed. The original transcripts were then re-reviewed to ensure the final themes were reflective of the data. The themes reflected the overarching HRQoL experiences during transitions; however, it was also noted that experiences were often shaped by modality specific nuances. These nuances were often experienced or emphasized differently across individuals, as such, it was not possible to synthesize common experiences. Examples from each modality were thus pulled from the data and highlighted descriptively based on HRQoL domains. No qualitative data software was used for data analysis.

Baseline characteristics were summarized with basic descriptive statistics. The KDQOL-36 surveys were scored using the scoring template provided by RAND corporation, which converts raw entry data to a score from 0 to 100 [16]. The additional domains from the KDQOL-SF were scored following instructions provided by RAND specific to these domains [16]. The mean and standard deviation were calculated for each of the KDQOL domains at baseline and at 3 months.

## Results

### Patient characteristics

**Fig. 1:**
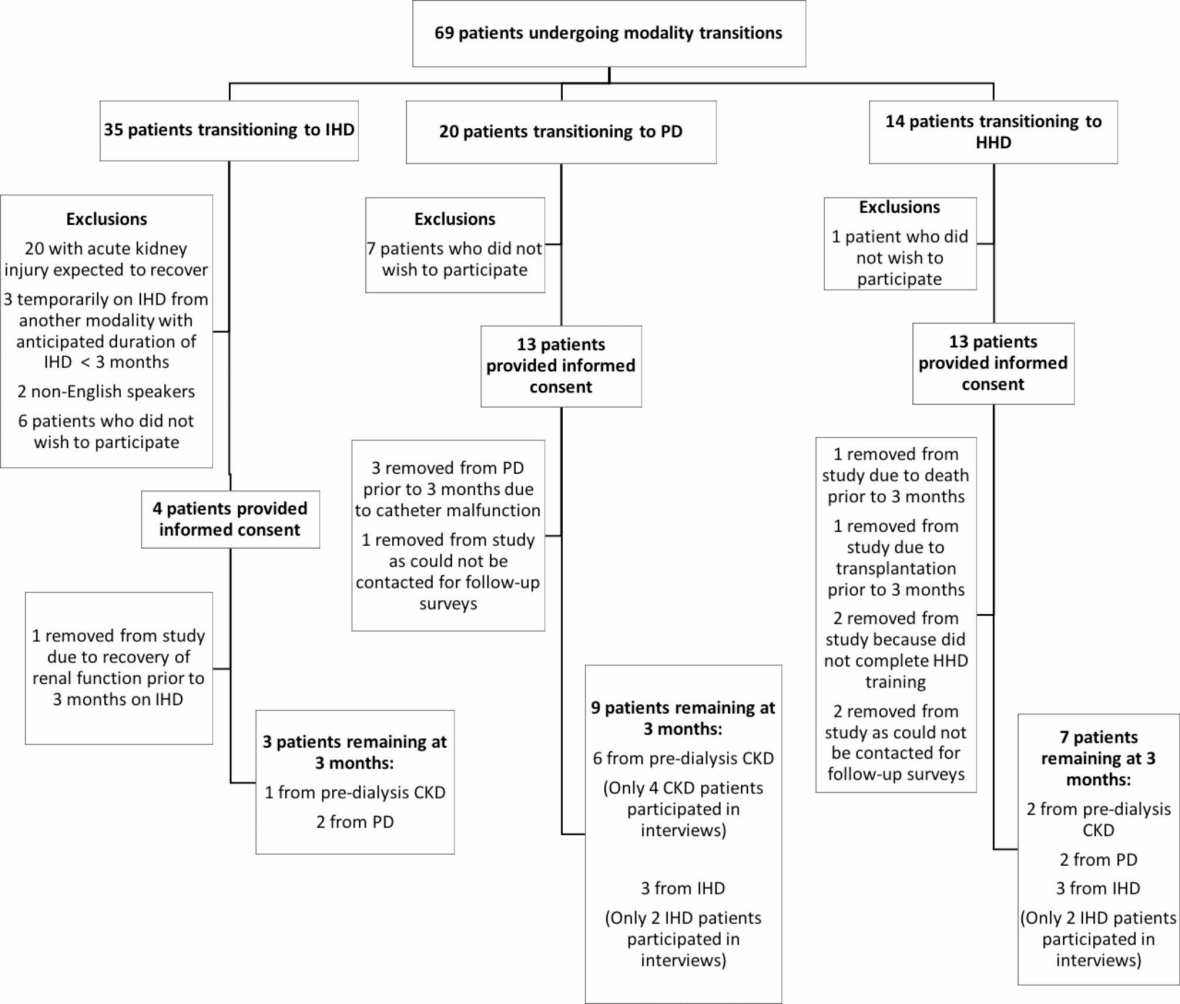
Patient Recruitment and Retention Depicts the patient flow for the study

Sixty-nine patients undertook modality changes during the recruitment period. Twenty-five were excluded based on pre-specified exclusion criteria. Of the 44 eligible patients, 30 consented to participate. Figure 1 shows the number of patients lost to follow-up. At three months, 19 patients (9 incident and 10 prevalent) remained in the study: 3 IHD, 9 PD, and 7 HHD. All 19 completed KDQOL surveys at baseline and 3 months. Four participants returned their surveys via mail but did not return our call to set up an interview. We attempted to contact these patients on three separate occasions, after which no further contact was made. Ultimately, fifteen participants were interviewed (range = 23 to 75 min). Table 1 shows the demographics of the 19 study participants. The mean age of the cohort was 50.9 +/- 16.9 years, with 73.7% male. Participants who were lost to follow up had similar characteristics. Dialysis vintage ranged from 0 months (incident patients) to one patient who had been on dialysis for over 7 years. All patients had hypertension and approximately one-quarter had diabetes. Other comorbidities were uncommon.

**Table 1:** Participant Characteristics

	Included Patients (n = 19)
**Age in Years, mean (SD)**	50.1 (16.9)
**Male, n (%)**	14 (73.7%)
**Dialysis Vintage in Months, mean (range)**	13 (0–86)
**Cause of Kidney Disease, n (%)**:	
*Diabetic Nephropathy*	2 (10.5%)
*Hypertensive Nephropathy*	3 (15.8%)
*Polycystic Kidney Disease*	5 (26.3%)
*Glomerulonephritis*	9 (47.4%)
**Comorbidities, n (%)**:	
*Hypertension*	19 (100%)
*Diabetes*	5 (26.3%)
*Coronary Artery Disease*	1 (5%)
*Congestive Heart Failure*	1 (5%)

### The transition experience

**Fig. 2:**
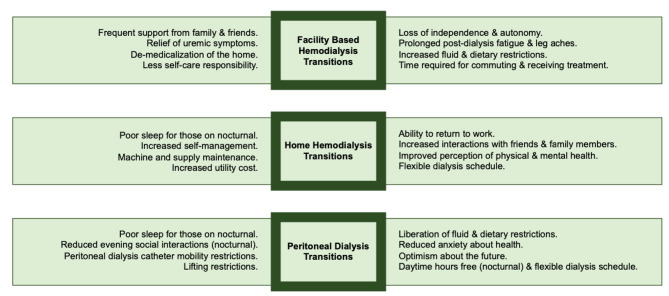
Nuanced factors that impact health related quality of life across dialysis modality type

Four overarching themes and five sub-themes were identified during the qualitative analysis that reflected the modality transition experiences of patients. Themes included: (1) adapting to new circumstances, sub-themes 1a) tackling change 1b) accepting change; (2) adjusting together; (3) trading off; (4) challenges of chronicity, sub-themes 4a) the impact of dialysis; 4b) living with a complex disease; 4c) and planning with uncertainty. Exemplar quotations for each theme are provided in Table 2. Participants further described the various ways in which modality specific nuances impacted their HRQoL during their transition (exemplars in Fig. 2; Table 3). An increase in mean KDQOL-36 scores were observed from baseline to three months on therapy within each domain of the KDQOL-36 regardless of where the patient was transitioning from (i.e. incident or prevalent patients) (Table 4).

**Table 2:** Health Related Quality of Life Scores from First Day on Dialysis to Three Months

KDQOL-36 Domain	All Patients (n = 19)			Incident Dialysis Patients (n = 9)			Prevalent Dialysis Patients (n = 10)		
	Baseline	3 month	Mean Difference	Baseline	3 month	Mean Difference	Baseline	3 month	Mean Difference
Symptoms/ Problems	61.9 (20.6)	76.8 (17.5)	14.9	62.2 (14.9)	81.0 (12.2)	18.8	61.6 (25.5)	72.9 (21.0)	11.3
Effects of Kidney Disease on Daily Life	49.0 (19.2)	63.3 (19.8)	14.3	50.7 (18.9)	66.7 (12.3)	16.0	47.5 (20.3)	60.3 (25.1)	12.8
Burden of Kidney Disease	32.2 (25.6)	39.5 (25.1)	7.3	29.2 (22.5)	36.1 (19.7)	6.9	35.0 (29.0)	42.5 (29.9)	7.5
PCS	32.0 (7.0)	39.4 (8.3)	7.5	31.0 (5.4)	39.7 (6.2)	8.7	33.0 (8.4)	39.2 (10.2)	6.2
MCS	42.4 (11.4))	49.6 (10.3)	7.2	38.7 (12.2)	48.6 (8.0)	9.9	45.7 (10.1)	50.4 (12.4)	4.7

Note: Baseline and 3-month scores represented as mean (confidence interval); All domains represent health related quality of life scores captured by the survey items from the Kidney Disease Quality of Life-36; Each domain is scored from 0 to 100, with higher scores representing better health related quality of life; PCS = physical composite score; MCS = mental component summary score; Incident dialysis patients = patients transitioning to a dialysis modality for the first time; Prevalent dialysis patients = patients who were already on dialysis but were transitioning to a different dialysis modality

**Table 3:** Exemplar Qualitative Quotes

Theme	Sub-Theme	Exemplar Quote
Adapting to New Circumstances	Tackling Change	I’m pushing [for decreasing water bills], first with our town and then maybe as a stepping stone to all of the cities and towns and municipalities… I think I’ve got it to where my town of XXX is going to start backing me up. [P4] If I eat too much of the wrong stuff, you realize it pretty quickly and you know you’re not going to feel right. But I mean, you just learn, you know, it’s your body. [P16] I’ve never been a person who sleeps in one position all night, so that’s going to be a significant challenge for me when I get to the point where I am using a fistula. [P5]
	Accepting Change	I think at the start, a lot of it was that I wasn’t working and I was sick and it was a lot to take in. So now, I know what’s going on and I have settled into my new routine… but, yeah, at first it was hard.[P6] I pretty much accept things as they are. It’s life. And, as I’ve been told, I am not 50…There’s no sense crying over spilled milk. [P26] I realize that I’m in a position where at least I have the opportunity to potentially receive a kidney transplant. I understand that lots of people aren’t in that position and that must really weigh on them….And I’m sure that would change my outlook if I knew I couldn’t get a transplant. [P15]
Adjusting Together		[My husband] had mentioned how helpless he felt in terms of being able to do anything to make my quality of life better and how frustrating it was for him that I never had energy to go out… He has commented since we started home dialysis that he feels that there is just way more that he can do to help. [P5] My fiancée has to put up with a lot, taking care of me and being responsible for the cooking and my moods and everything. And not being able to travel together. She’d like it if we could travel together, and so would I. But it’s just not in the cards right now. [P18] [My wife] feels she has to be here in the house all the time in case something goes wrong… I’m trying to reassure her that she can [travel] and she doesn’t have to worry about me, because I’ll be fine. [P4]
Trading Off		I have more energy and a lot more drive, I think, since I switched from the hemo to the peritoneal. I think it’s mostly that I didn’t have to go to the hospital every second day. [P29] Not going to the hospital… saves us money on things like parking and gas. So, that’s a positive point for me. The other one is that, you know, I get up in the morning and I’m already done with dialysis. I mean, not so much right now because I’m doing an extra bag in the middle of the day, but basically most days I’m done with it… So I think there are more positives with peritoneal dialysis compared to hemodialysis. [P8] Apparently peritoneal dialysis doesn’t get rid of calciphylaxis. The only way you can get rid of it is on [peritoneal dialysis]. [P26]
Challenges of Chronicity	The Impact of Dialysis	I’d like to be able to drink, you know, instead of having to eat ice chips. I drink a couple of cups, 2 or 3 cups a day, but I’d like if it was more (coughs). And I’ve got a cold now. Probably because my immune system is so suppressed. [P18] I had to get two catheters put in. The first one had to be removed… So, I had another month and a half where I couldn’t go to work and then in my industry… when the job comes up you’ve got to take it. There’s only so many jobs. [P19] I play more games now. I play a lot of cards again. I play Bingo. The only problem is I cannot go to places overnight because of dialysis in the evening. [P9]
	Living with a Complex Disease(s)	I’ve been sick for a year and a half now so I haven’t really broken a sweat in a year and a half. I’ve lost a lot of my muscle in that time…. And my physical health has declined because of that. [P15] I mean, it is all connected [diabetes and kidney disease]. Unfortunately, we just can’t treat one part of the body and expect the rest of the body to stay as it was, right? [P5] My vision has actually gotten worse lately. I’m going for eye tests next week. And also, my feet are really, really sore. Now, the doctor has given me some pills to try to counteract that, but as the day goes on, my feet get really sore. [P4]
	Planning with Uncertainty	It’s just that I’m not used to it and I wasn’t expecting the whole situation and all of a sudden, everything has fallen in my lap, and I’ve had no choice but to deal with it. [P8] Some days are better than others. So, some days I can work all day long, whereas other days, a half a day is all I can do. [P6] Sometimes I can do everything in my house but there’s times that I can’t. That’s why I have a caregiver. I easily get tired. [P11]

**Table 4:** Nuanced factors that impact health related quality of life across modality type: Exemplar quotes

Domain	Starting In-centre Hemodialysis	Starting Home Hemodialysis	Starting Peritoneal Dialysis
**Physical health and well-being**	“When I’m on hemodialysis, I’m *always* tired. If I wasn’t on dialysis, I wouldn’t be tired…I just don’t have the energy anymore.” “Before I started hemo, I couldn’t get out of a wheelchair, I couldn’t get out of a chair. Somebody had to help me into bed and out of bed and get my clothes on and have a shower….but I’m getting stronger.”	“I have a lot more energy and I am a lot less tired. I generally just don’t have the kind of fatigue that I would get after an in-centre dialysis treatment, and I don’t feel as fatigued on the few days that I do take off.” “They were able to take me off pretty much all of my blood pressure medications. So I’m only on one pill now, and it’s a lower dose than what I was originally on.”	“I find myself more sluggish with all the fluid in with the PD. I’m back to work now and I just feel sluggish.” “I can do many things that I could not do before….I used to not be able to go up the stairs without shortness of breath….now I walk to [the grocery store]. It is about 3 kilometres from my house. We walk there now.”
**Mental health, well-being, and self-perception**	“I know that there is a part of me that is never going to get better….I pretty much accept things as they are. It’s life.” “Lower energy. Getting depressed. You just don’t even know what you can do. I was hoping I would go from hemo to PD to transplant. But now that PD has failed, I feel like I’m starting all over again”.	“I think overall I am just more independent, more in-tune to how I am feeling. I am more aware of my health, and I am just better educated about dialysis and how it is helping me.” “I feel like I have a lot more hope. I feel hopeful that I can have some sort of quality of life.”	“I used to worry about the future and what would happen to me. I thought I should go back to the Philippines because I did not have much life left. But since dialysis, I feel better.” “Sometimes I get irritated by things. It’s a big change in lifestyle and I guess that’s what’s getting me down more than anything else. But it’s getting better. The PD is helping.”
**Interactions with/impact on family and friends**	“As far as support, everyone has just been great. You know, my daughter goes to work a half hour early in the morning and stays a half hour later at night so that she can take an hour at lunch to take me to dialysis. She’s just been such a huge help.” “They’re afraid to let me do anything. I have to tell them, ‘You know, I can do some things. I *need* to do something’….nobody has asked the doctor if I should just sit on my chair all the time.”	“I’m spending more time with them [family]. And…I think they’re more in-tune with what I am doing as well, because it’s more front and centre and visible to them.” “There is some impact on my wife. She feels she has to be here in the house all the time in case something goes wrong…I’m trying to reassure her that she can [leave the house] and she doesn’t have to worry about me, because I’ll be fine.”	“I don’t visit with friends as much because I go to bed earlier.” “My wife, and sometimes even my children, they don’t want me to participate in things that they think are not proper for me…for example, sometimes washing dishes, my wife tells me that if I get myself wet, especially where the tube is coming out of my belly…it might start an infection and I might get in trouble. Therefore she tells me not to touch anything.”
**Symptoms of kidney disease/dialysis**	“I find that most days after dialysis, I have a lot of soreness in my legs….they call it restless legs, but mine are sore. It’s not that they have to move. It’s that they ache.” “My legs get achey after dialysis, usually from my knees down…it’s not cramps. It’s different from cramping. It’s aches.”	“I don’t get the cramps. I don’t get the itchiness. All of the nausea and throwing up and all of that stuff is gone.” “I do feel much better now, on home hemo, even though I have headaches that last for 5 to 6 hours after I have finished dialysis. I would choose that over the total sleeplessness, and the drain pain, and the brain fog that I had on peritoneal dialysis.”	“The itchy skin is gone….it was driving me crazy [before PD].” “I get heartburn like you wouldn’t believe, with all the fluid in there….I’ll have chewed up half a dozen Tums just to get to the end of the day. Twenty-four hours a day I got heartburn. Nighttime is usually the worst.”
**Quality of sleep**	“I was getting up a lot in the middle of the night to go to the bathroom before dialysis….not so much after I started. I was peeing less.” “On PD, I hooked up and went to bed, and then I got up in the morning. Very seldom was I awake all night. But with this [IHD], I can be awake a lot.”	“Some nights, for whatever reason, my machine alarms a couple of times, so obviously those nights I get a little less sleep. But that’s not really that often, and overall my sleeps haven’t been impacted at night.” “I would say that in his [husband] sleep quality, it has impacted us negatively. But in terms of my quality of sleep, I do sleep better on home hemo that I did on peritoneal because I don’t have the pain during the night.”	“At the beginning…I turned and tossed in the bed, and the tube would get twisted and cut off the flow and then the machine would start making all kinds of noises, so that was a bit of a challenge. But slowly I’m getting used to it.” “I still haven’t adjusted to the night, because when the cycler changes and starts to drain like a vacuum, I just sort of wake up…but I am adjusting and it’s getting a little better over time.”
**Ability to maintain employment**	“I had more energy at work [when on IHD] because I was sleeping better.”	“Because I just feel better at work, I think that my productivity while at work has improved.” “I wasn’t working while I was on PD. I just went back to work after the home hemo started…For the most part, I work half days.”	“As far as working goes, with my specific job, I don’t have a problem with it….I can go to work and I can provide for my family. I can do that….if I had to do hemo well…I couldn’t do what I do for a living.” “I was a farmer, so I’m quite physical, but I can’t really do that kind of work anymore like I used to. If I had had a difference source of employment, I probably could have been just fine.
**Participation in hobbies, activities, and travel**	“Now when you want to do something, you have to say ‘Well, let’s wait and see where I am in the morning’. Whereas with the peritoneal dialysis, I could just do. Sometimes I’d be tired, but I’d be able to do it.” “And being trapped in Calgary. Because I can’t go anywhere. Well I can, for maybe two days, but I have to be back right away. And I have to go somewhere where there’s a machine”.	“Say if we wanted to go somewhere and stay somewhere else overnight, we have that possibility now.” “I mean, basically these machines place you on house arrest. I can go away for a couple of days, but I have to make sure I get back in time to get onto the machine on time, otherwise I’m going to be in big trouble.”	“I used to brush my teeth and then be gasping for breath. Now that doesn’t happen. Now I play ping pong on Sunday nights. And billiards downstairs. I want to do everything.” “It ruins my social life, because I have to wake up at 7 o’clock [to do a manual exchange] and then do it again at 11 o’clock and 3 in o’clock in the afternoon and then 7 o’clock at night, so I don’t go nowhere.”
**Overall changes to health-related quality of life**	“The big thing is the time involved in getting here, getting the four hours of treatment, and going home.” “It’s not the best, but I guess it’s all I’ve got right now until hopefully a transplant comes along. Six to ten years is a long time to wait though. But I guess I’m stuck with this for now.”	“I think there’s flexibility and it’s one of the best things about home hemo. You can pretty much be a master of your own time, you know, and do it however you like.” “It’s considerably more work to be on home hemodialysis, but I think the results, in terms of my health and how I feel, are worth the extra work.”	“I have a lot more energy and a lot more drive, I think, since I switched from the hemo to the peritoneal.” “I get up in the morning and I’m done with dialysis….I don’t have to think about it or worry about it. I don’t have to spend four or five hours going on the road to the hospital and getting my treatment then coming back. So I think there are more positives with peritoneal dialysis compared to hemodialysis”.

#### Theme 1: adapting to new circumstances

*1a. Tackling Change.* Dialysis care trajectories are not static processes, and often transitions did not go smoothly or as planned. Participants spoke of the ways in which they overcame both routine and unexpected changes and challenges in order to fit their new modality into their life. Some participants tackled change in active ways by advocating for unmet needs or adjusting the environment around them.

*When I first got set up and everything, you walk into the room and I was like man, this reminds me of a hospital room. But now, it’s just contained to the bedroom. So it’s fine. [P6]*

Others transitioned in a more passive way as the environment around them seemed outside of their control. This process was often cyclical where participants would “figure things out” yet often also be wary of what changes and challenges were to come.

*1b. Accepting Change.* Accepting change was discussed by participants in regard to the intricacies within both starting dialysis and the health changes that came with worsening complications of kidney disease. Though most participants learned to adjust to their new circumstance as their new treatment method was necessary, they experienced different degrees of acceptance at the time of interview.

*I still haven’t adjusted to the night, because when the cycler changes and starts to drain like a vacuum, I just sort of wake up and I’m not quite sure why. But I am adjusting, and it’s getting a little better over time. [P29]*

Some participants compared their situation to better times and spoke of what had been lost (e.g. physical functioning, ability to work). While others focussed on the positive ways in which their new modality improved their life (e.g. symptom improvement, treatment flexibility). The degree of acceptance was often influenced by individual contexts and views including their medical status, where they were transitioning from, their age, their work status, their interactions with other patients, and their future prospects (e.g. transplant).

#### Theme 2: adjusting together

*My wife is sort of limited to falling asleep when I am finally set up and limited to waking up when I wake up as well. So, she’s also not getting the best sleep even when she is sleeping. [P1]*

Participants spoke of the ways in which their kidney disease and transitioning to dialysis or a new modality impacted both their lives and the lives of those around them. Some participants spoke of this as a positive change, where they were feeling better and able to be more present, or their new modality was better suited to family dynamics. Other patients spoke of how they felt like a burden to friends and family and felt guilty about the impact their disease had on the people in their lives. Regardless, everyone was adjusting through this process together. Responsibilities also often needed to be adapted based on everyone’s needs, not just of those of the patient. At times there were conflicts in roles between what the patient felt they needed and could do, to what their support person felt they needed or should be doing.

#### Theme 3: trading off

*… I’m feeling better compared to before. Other than actually having to go on dialysis at night. I am feeling better. [P6]*

Most participants spoke of a give and take that occurred on dialysis and during modality transition. Having kidney disease was far from perfect and it was clear that there was no “best” dialysis treatment. As such, participants spoke of a trade off, giving up some things in favor of others. For example, one man spoke of how even though home dialysis was a lot of work to set up, it was better than driving to a facility three times per week. Ultimately, participants spoke of finding the modality that suited them best. This was a dynamic process, and as patients’ needs and circumstances changed, so did this “trade off” and subsequent modality fit. At times, the reason for modality switch was out of their control and patients could no longer stay on their intended therapy (i.e., PD membrane failure).

#### Theme 4: challenges of chronicity

*4a. The Impact of Dialysis.* Most participants expressed an improvement in some symptoms of kidney disease after their transition. However, the participants responded to their dialysis therapy differently and there were various other elements that impacted their HRQoL. The perceived impact of dialysis was important to understand in the context of how their dialysis modality met their expectations and what they were prepared for or were able to manage (e.g. financial impact of not working or time traveling to dialysis).

*It ruins my social life, because, I had to wake up at 7:00 am to do [manual exchange of PD solution] and then 11:00 again and then three o’clock in the afternoon and then seven o’clock at night, so I don’t go nowhere… Even my groceries, I have to make it quick because I don’t want to be late for my exchange. [P11]*

*4b. Living with a Complex Disease(s).* Patients were often not only living with kidney disease, which was already difficult enough, but frequently patients had additional comorbidities. This resulted in constant changing needs and symptoms that ebbed and flowed as their disease progressed. The impact was felt in all aspects of the patient’s life and appeared to vary based on what life stage the participant was at.

*Since I got sick I cannot work anymore…with the kind of job I had, you have to be standing, running around, I cannot do that anymore. I easily get tired. [P11]*

*4c. Planning with Uncertainty.* Living with kidney disease among other comorbidities led to feelings of uncertainty and often a lack of control. For some participants this was difficult, as it was hard to plan for the future when how they felt and where they were (e.g. dialysis modality, in hospital) could change quickly. However, many patients took things “day by day” and did not feel bound by the constraints felt by others. This enabled them to feel hopeful for the future and excited to plan for future events.

*I still don’t feel that I have enough trust in my ability to commit to being able to do something…over today, but I’m not too sure what tomorrow will bring. [P5]*

## Discussion

We identified four primary themes that may help us understand the patient experience during their transition to a new dialysis modality: adapting to new circumstances, adjusting together, trading off, and challenges of chronicity. Overall, our findings illustrate that modality transitions represent a time of significant change that can be difficult for patients regardless of what modality they are transitioning to, or from and regardless of if it was an incident or prevalent transition. This reinforces the importance of creating integrated models of care that support people during transitions across the kidney disease trajectory [24].

We found that from the first day of dialysis treatment to the third month on a new dialysis therapy, all five HRQoL domains from the KDQOL-36 (symptoms, effects, burden, overall PCS, and overall MCS) improved in our sample (i.e., those who remained on the modality). Although we were underpowered to assess for statistical associations, we noticed that KDQOL-36 scores were similar across both incident and prevalent dialysis transitions, potentially indicating that dialysis transitions may negatively impact HRQoL regardless of where an individual is in their care trajectory. HRQoL scores are known to be lower in people on dialysis than the general public, as well as those who have received a kidney transplant, making HRQoL an important element in care for people on dialysis irrespective of dialysis modality choice or transition time period [25, 26]. However, the low HRQoL scores at the beginning of the transition period in our study demonstrate that the early transition period may be a particularly difficult time that would require supportive interventions.

Although our small sample size made it inappropriate to ascertain clinical meaning or statistical associations from the HRQoL scores in our study, the qualitative findings reveal the turmoil that may be present during dialysis transitions. Participants in our study spoke of how they had to tackle constant changes and challenges, adapt to their new roles and responsibilities, and adjust to the intricacies of each modality therapy, all while managing a complex illness. This was true across both incident and prevalent transitions. It is thus not surprising that other authors have reported that 44% of people initiating dialysis meet the criteria for depression [27]. A qualitative study of 36 people initiating hemodialysis identified three major constructs that patients describe during a transition period: “redefinition of self”, “quality of supports”, and “meanings of illness and treatment” [11]. In particular, the perceived quality of supports had a large impact on how people redefine themselves during transitions [11]. It is thus possible that providing adequate support during transition periods may positively impact HRQoL.

Interestingly, how patients described the transition period in our qualitative interviews differed, with some patients comparing their current situation to the last time they were feeling well on either a previous dialysis therapy, before they started dialysis, or even before they got sick with kidney disease, while others described the transition as the day of dialysis start. This was also true for when, and often if, their transition period ended. This creates further challenges as it makes it difficult to ascertain how long supportive interventions are required for, or when they are most effective at easing transition burden.

Individualized care is challenging in current dialysis facilities, where resources for patient-specific supports are often limited by time and budgetary constraints. Despite growing recognition that transition periods are times of immense difficulty for patients, relatively little is known about methods to ease physical and emotional strain during transitions. Clinicians often focus on medical complications during transition periods rather than psychological complications, and some experts have suggested psychosocial and spiritual factors should be attended to more by dialysis staff during transition states [10, 28]. Some authors have even suggested that transitional care units should be standard of care; in these units, patients would receive more individualized care for the initial weeks to months of dialysis, allowing a more intensive focus on their individual motivations and goals [29, 30].

Clinically, our findings highlight the importance of focusing on supporting patients during dialysis transitions, regardless of modality vintage. Viewing the transition experience from a more holistic lens may enable kidney programs to put common interventions in place across all phases of the care trajectory and ultimately better support patients across programs rather than in siloed care structures and dialysis units. Future research should aim to identify individual patient needs during transitions and to implement effective interventions to meet them. For interventions to be effective, a better understanding from patients and their families about the breadth and depth of the different meanings of and definitions attributed to transition periods are needed.

While our study yields important findings regarding the patient experience during transition periods, there are some important limitations. First, our recruitment period was short, yielding a small number of patients transitioning to and from a variety of dialysis vintages. This source of heterogeneity may have influenced our results. Statistical testing was not performed due to small sample size. However, despite our small sample size we achieved thematic saturation with our interviews. The fact that non-English speakers were excluded from our study is an important limitation, as it stands to reason that lacking the ability to fully communicate with care providers may have a profoundly negative impact on quality of life during the transition period. It is also possible that the true HRQoL scores during dialysis transitions are lower than what we found since many people who were approached to participate in our study refused as they were too overwhelmed by their circumstances. Participants who were lost to follow up also most often either died, or experienced peritoneal dialysis catheter malfunction sparking another modality change, both which may have further negatively impacted HRQoL. Although large difference in HRQoL were not noted between incident and prevalent dialysis starts, this would be an important avenue to explore in future work. Lastly, our sample was predominantly male, and sex and gender differences may impact transition experiences.

## Conclusions

Modality transitions represent a time of significant change that can be difficult for patients regardless of what modality they are transitioning to, or from. Future research should focus on exploring this transition with larger sample sizes and in different contexts with a goal of seeking insight into how transitions can be improved.

### Electronic supplementary material

Below is the link to the electronic supplementary material.

Supplementary Material 1

## Data Availability

We are not able to make our dataset publicly available due to the potential to identify participants in both the qualitative and quantitative data. Please contact the corresponding author should you wish to discuss this further.

## Abbreviations

HRQoL: Health related quality of life
IHD: In-center hemodialysis
HHD: Home hemodialysis
PD: Peritoneal dialysis
KDQOL-36: The Kidney Disease Quality of Life-36 (KDQOL-36)
PCS: Physical component summary score
MCS: Mental component summary score
MCID: Minimal clinically important difference
