# Supplementary material for: Health related quality of life during dialysis modality transitions: a qualitative study
Source: BMC Nephrol. 2023 Sep 22;24:282. doi: 10.1186/s12882-023-03330-y (PMC10517513; doi:10.1186/s12882-023-03330-y)
Supplement: Supplementary file 1 — Supplementary Material 1 [file 12882_2023_3330_MOESM1_ESM.docx]

**Qualitative Interview Script**

*Hello, my name is _______, and I am one of the researchers from the Southern Alberta Renal Program. I am leading the dialysis transitions study that you are participating in. This is one of the scheduled phone calls that was discussed when you signed up to participate in this study. I would like to ask you a few questions about your experience with dialysis so far. Do you have time to answer a few questions right now?*

*Before we begin, I just want to remind you that these questions are only being used for the study. Your responses are confidential and will be made anonymous as soon as this phone call is completed. This phone call will be recorded so that we can review your answers if we need to as the study moves forward.*

*I’m interested in learning about your experience receiving a new modality of dialysis. I would also like to know about any changes that have occurred in your daily living since starting your new dialysis therapy.*

***First Question:***

*To begin, can you describe any changes in your quality of life that immediately come to mind? These can be either positive changes or negative changes.*

- Depending on the response, use open-ended questions to prompt for more information:
  - Can you tell me more about that?
  - What impact do you think that has had?

***Second Question:***

*Now there are some specific areas of quality of life we’d like to explore, and I’m interested in your thoughts on each of these areas. How do you think your new dialysis therapy has impacted your life in terms of:*

*a) Physical health and well-being (for example, energy levels, ability to perform physical tasks, ability to get things done)*

*b) Mental health and well-being*

*c) Interactions with family and friends*

*d) Symptoms of kidney disease or side effects of dialysis – have you noticed anything that’s gotten particularly better or worse on your new therapy?*

*e) Sleep*

*f) Ability to work*

***Third Question:***

*Could you tell me about any concerns related to the impact of your dialysis therapy on your family members or other caregivers?*

- If changed dialysis modalities, ask about a comparison to previous modality.

***Final Question***

*Are there any other comments or concerns you have for us before we end our conversation today?*

*That’s all the questions I have today. Thank you very much for participating in this phone survey.*
